# ﻿Phylogenetic review of the millipede genus *Cherokia* Chamberlin, 1949 (Polydesmida, Xystodesmidae)

**DOI:** 10.3897/zookeys.1106.81386

**Published:** 2022-06-20

**Authors:** Luisa Fernanda Vasquez-Valverde, Paul E. Marek

**Affiliations:** 1 Virginia Tech, Department of Entomology, 170 Drillfield Drive, Blacksburg, Virginia 24061, USA Virginia Tech, Department of Entomology Blacksburg United States of America

**Keywords:** Citizen science, DNA barcoding, morphology, phylogenetics, subspecies

## Abstract

The millipede genus *Cherokia* Chamberlin, 1949 is a monospecific taxon, with the type species *Cherokiageorgiana* (Bollman, 1889). The last revision of the genus was made by Hoffman (1960) where he established three subspecies. Here we used molecular phylogenetics to assess the genus and evaluate whether it is a monophyletic group, and if the subspecies are each monophyletic. We included material from literature records and three natural history collections. Newly collected samples were obtained through a citizen science project. Morphological characters underlying subspecies groups—the shape of the paranota, body size, and coloration—were evaluated. A molecular phylogeny of the genus was estimated based on DNA sequences for seven gene loci, and a species delimitation analysis was used to evaluate the status of the subspecies. The documented geographical range of *Cherokia* in the United States was expanded to include a newly reported state record (Virginia) and about 160 new localities compared to the previously known range. Morphological characters, which included the shape of the paranota and body size that had been historically used to establish subspecies, showed clinal variation with a direct relationship with geographical distribution and elevation, but not with phylogeny. Coloration was highly variable and did not accord with geography or phylogeny. The phylogeny recovered *Cherokia* as a monophyletic lineage, and the species delimitation test supported the existence of a single species. The subspecies *Cherokiageorgianaducilla* (Chamberlin, 1939) and *Cherokiageorgianalatassa* Hoffman, 1960 have been synonymized with *Cherokiageorgiana*. The molecular and morphological evidence showed that *Cherokia* is a monospecific genus with the sole species, *Cherokiageorgiana*, being geographically widespread and highly variable in its morphology.

## ﻿Introduction

**Figure 1.**
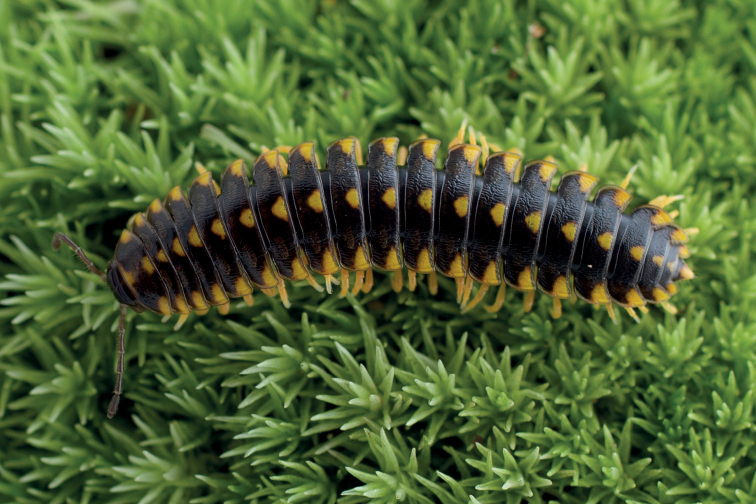
*Cherokiageorgiana* (Bollman, 1889), the wrinkled flat-backed millipede. Dorsal view of the whole body of specimen MPE04539 (Male, GA – White Co.) deposited in the Virginia Tech Insect Collection (VTEC). The image shows the more common coloration for the species and the prominent wrinkles of the cuticle.

The family Xystodesmidae (Polydesmida) includes 539 species with a center of diversity concentrated in the Appalachian Mountains (Means et al. 2021a, b; Hennen et al. 2022). Within the family Xystodesmidae, the Appalachian genus *Cherokia* Chamberlin, 1949 (Fig. 1) was named after the Cherokee, an indigenous group of peoples in the southeastern United States. This monospecific genus in the xystodesmid tribe Rhysodesmini was erected by Chamberlin (1949) for the species *Fontariageorgiana* Bollman, 1889 as its type species. After its description, various authors proposed multiple species that were all subsequently synonymized with the type species *Cherokiageorgiana* (Bollman, 1889) based on gonopod morphology (Causey 1950; Hoffman 1950; Chamberlin and Hoffman 1958). However, all the above-mentioned authors pointed out the considerable color, body size, and shape variation in millipedes of the genus *Cherokia*.

**Figure 2.**
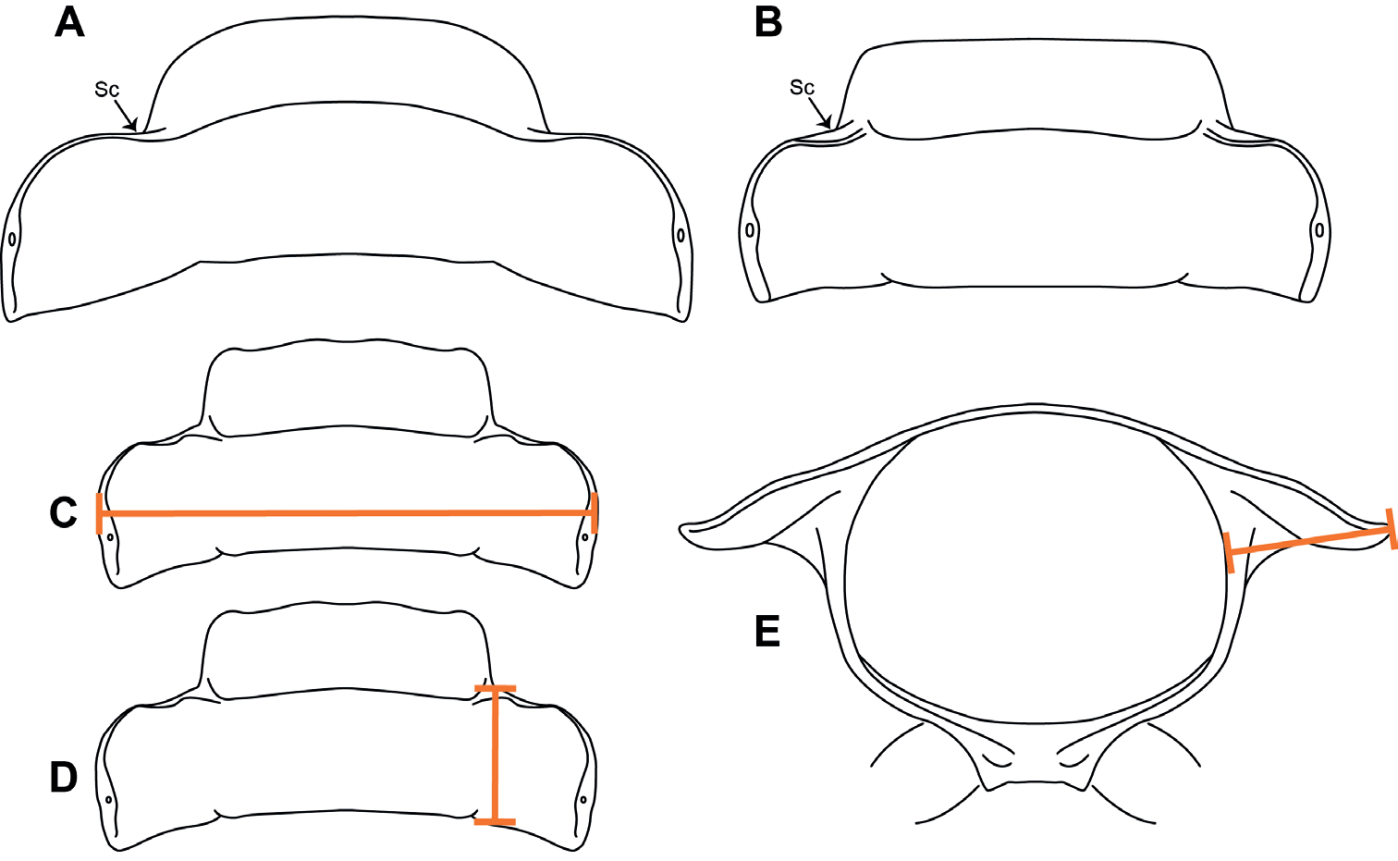
Position of the scapulorae (Sc) **A** strictly marginal **B** submarginal. Measurements of the 12^th^ body ring **C** metazonite width **D** metazonite length **E** paranota extension. Adapted from Hoffman (1960).

Prior to Hoffman’s (1960) revision, no one had carried out a comprehensive synthesis of this genus. He (Hoffman 1960) proposed *Cherokia* as a monospecific genus, with the sole species *Cherokiageorgiana* divided into three subspecies: *Cherokiageorgianageorgiana* (Bollman, 1889), *Cherokiageorgianaducilla* (Chamberlin, 1939), and *Cherokiageorgianalatassa* Hoffman, 1960. Hoffman (1960) described in detail the morphological variation and geographical distribution of *Cherokia*. He also differentiated the three subspecies from each other based on morphological features that included the position of the scapulora and the ratio of the body length versus its width. The scapulora is a term defined by Hoffman (1960: 231) as: “from the Latin “*scapula*,” a shoulder, and “*ora*,” the rim of a shield”. The scapulora in *C.g.latassa* is found in a marginal position, which separates it from *C.g.georgiana* and *C.g.ducilla* that have a submarginally located scapulora (Fig. 2A, B). The subspecies, *C.g.georgiana* and *C.g.ducilla*, were differentiated from each other based on the ratio of the previously mentioned body measurements (i.e., body length versus its width) (Hoffman 1960).

Hoffman (1960) confronted various problems during his revision of the genus *Cherokia*. The first one, he explained, was the fact that “despite the diversity of body form, color pattern, and morphological details which occurs in the genus, the male gonopods remain essentially similar” (Hoffman 1960: 227). Although some variation in the solenomere shape in specimens in the North Carolina mountains was observed by Hoffman (1960), the character was not consistent and did not correlate with geographical distribution or subspecies differentiation. Additionally, the same author expressed a struggle with confidently assigning all individuals to one of the subspecies. For this reason, Hoffman (1960) proposed an intermediate form, termed an “intergrade” between *C.g.georgiana* and *C.g.ducilla*. These intergrades were documented within a wide geographical band (~30 km) in western North Carolina between the distributions of *C.g.georgiana* and *C.g.ducilla*.

After 1960, some authors have indirectly mentioned *Cherokia* in tribal revisions (Hoffman 1978) and checklists (Shelley 1980, 2000; Marek et al. 2014). More recent research on the family Xystodesmidae, using a synthesis of morphological and molecular characteristics, has confidently placed the genus *Cherokia* within the family Xystodesmidae and subfamily Rhysodesminae Brolemann, 1916 as sister to the genus *Pleuroloma* Rafinesque, 1820 (Means and Marek 2017; Means et al. 2021b). As a result of field collections for this recent work, a large number of *Cherokia* individuals were collected from throughout the eastern United States, and within its range. These recent results combined with materials from natural history collections from the mid-1900s up to now, point to an even greater diversity than initially uncovered.

Here we used natural history collections in combination with new material sampled from nearly 200 locations within the range of *Cherokia*. These new samples, specially prepared for preservation of DNA, provided the basis to estimate an evolutionary history using molecular phylogenetics and address the status of the three subspecies within *Cherokiageorgiana*.

## ﻿Materials and methods

### ﻿Selection of samples and Citizen science project

Specimens of the genus *Cherokia* preserved in the Virginia Tech Insect Collection were selected based on the availability to score both morphological and molecular characters from them. Individual live millipede specimens or their tissues were fixed in either 100% ethanol or Qiagen RNAlater thereby preserving DNA and other genetic material. Whole body specimens (minus tissue preserved for DNA) were then preserved in 70% isopropanol for subsequent morphological evaluation.

New samples were needed from some localities that had not previously been sampled; these localities were in the periphery of the known distribution of *Cherokia* or in areas where DNA-grade specimens were unavailable. A season of fieldwork was planned for the Summer 2020, however, due to the SARS-CoV-2/COVID-19 pandemic, and state restrictions, travel was not feasible. In response, and with the objective of obtaining these required samples, a citizen science project was developed. This enabled the general public to participate in the collection of millipedes of the genus *Cherokia*, and to learn about biodiversity.

**Figure 3.**
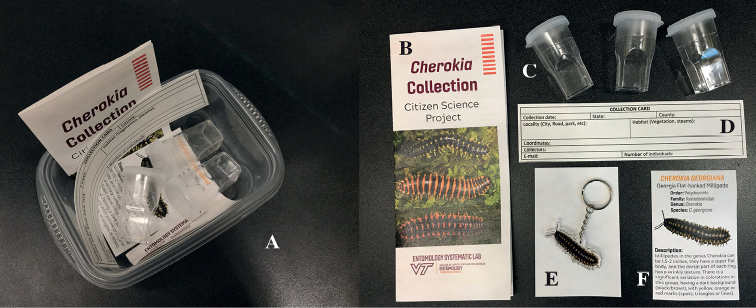
Citizen science collection kit **A** plastic food container (32 FL OZ) **B** instruction flyer: with step-by-step instructions of collecting and shipping **C** clear plastic collection vials **D** collection card **E** token for the participant: millipede keychain and **F***Cherokia* identification card.

For the citizen science project, collection kits and information pamphlets were designed with step-by-step instructions and other information for the public to obtain samples in an accurate and lawful way (Fig. 3B). Citizen scientists were recruited with social media through Facebook and Twitter, and the kits were shipped to interested participants. A small plastic keychain of *Cherokia* was included in the kit as a token of participation (Fig. 3E). Once the participants received the kit and collected millipedes, they were instructed to ship the millipedes to the lab at Virginia Tech, so we could identify, process and preserve them following methodology described by Means et al. (2015).

### ﻿DNA extraction, phylogenetics analysis and species delimitation

Preserved tissue (legs or head) from each individual was used for DNA extraction with a Qiagen DNeasy kit. The DNA obtained from the extraction was amplified via polymerase chain reaction (PCR) for seven gene regions: cytochrome oxidase subunit I (COI), small subunit RNA (12S), tRNA-Valine (tRNA-Val), large subunit RNA (16S), elongation factor-alpha (EF1α), RNA polymerase II largest subunit (RNAPol2), and F-box (fBox). The mitochondrial 12S, tRNA-Val, and 16S regions were amplified as a single contiguous stretch. Amplification of DNA was carried out according to Means et al. (2021a, b). These PCR amplicons were cleaned, quantified, normalized, and sequenced on an Applied Biosystems ABI 3730 capillary sequencer at the University of Arizona Genetics Core.

The sequences were analyzed in Mesquite (Version 3.61) (Maddison and Maddison 2019) using the sequence analysis module Chromaseq (Version 1.52) that implements phred and phrap (Ewing et al. 1998; Maddison and Maddison 2020) for chromatogram base calling, trimming, quality control and generation and curation of matrices. The outgroups were selected based on the phylogeny inferred by Means et al. (2021b) and included a single individual of: *Pleurolomaflavipes* Rafinesque, 1820, *Pleurolomaplana* Shelley, 1980 and *Pleurolomacala* (Chamberlin, 1939). Sequences were aligned with the progressive sequence alignment program MAFFT (Version 7) using the model L-INS-I (Katoh and Standley 2013). A nucleotide base composition homogeneity chi-square test in IQ-TREE 2 (Version 2.0.4; Minh et al. 2020) was run with the aligned sequences for each of the genes to test the heterogeneity of the sequences (H_alternative_ = homogeneity) and excluding the sequences of the outgroup taxa. The sequences that failed the heterogeneity test were excluded from the phylogeny. Afterwards, each locus was partitioned by gene, intron/exon location, and codon position. The seven loci were concatenated into a single matrix. The partitioned matrix was analyzed using ModelFinder to test alternative nucleotide evolution models and to infer the model of best-fit for the data (Kalyaanamoorthy et al. 2017). The selected model was then used to estimate a phylogenetic tree for the genus with the maximum likelihood-based phylogenetics software IQ-TREE 2. Single gene alignments were then analyzed separately to estimate gene trees with the same methods and software as above.

To determine whether the subspecies of *Cherokiageorgiana* represent distinct genetic groups, Automatic Barcode Gap Discovery (ABGD) species delimitation analysis was used. This method uses an alignment of sequences of a single locus (COI) to make a pairwise distance matrix and determine if a barcode gap exists. A barcode gap is observed when the intraspecific distance among unique sequences is smaller than the interspecific distance (Puillandre et al. 2012). This analysis was run in the ABGD online server using the alignment of *Cherokia* sequences for the locus COI, excluding the outgroup sequences.

### ﻿Distribution mapping and morphological characters analysis

To infer a detailed geographical range of the genus *Cherokia*, records in the literature, natural history collections, and new collections from the citizen science project were included. All the localities of specimens of *Cherokia* documented in Hoffman (1960) and from the Virginia Tech Insect Collection, Virginia Museum of Natural History (VMNH), and Florida State Collection of Arthropods (FSCA) were digitized. Digitization involved transcribing the label data of specimens in a spreadsheet using the Darwin Core data standard (Wieczorek et al. 2012). Text-based details of the label including state, county, and any other locality information were manually entered in a spreadsheet. In cases where precise geographical coordinates (e.g., latitude and longitude) were not provided, the text of the localities from the labels was transcribed, georeferenced and geographical coordinates automatically estimated using the software GEOLocate (Rios and Bart 2010) to retrospectively obtain decimal degree coordinates and an error radius based on precision of the text locality. To supplement this data set, localities from *Cherokia* specimens from the Virginia Tech Insect Collection (VTEC) that were already digitized and with geographical coordinates recorded at the time of collection were downloaded from the online database SCAN (Barkworth et al. 2019). This data set of coordinates (from collections and literature), was the basis to produce a comprehensive map of the geographical range of *Cherokia*. The map was constructed in the online GIS application SimpleMappr (Shorthouse 2010).

For the analysis of morphological features, the traits described in Hoffman (1960) were revisited: width-to-length ratio, color (hue and pattern), gonopods, and the position of the scapulora (Fig. 2A, B). Hoffman (1960) measured the entire length of the trunk of the millipedes; however, due to the flexibility of the trunk and the rings that make up the trunk—causing accordion-like compression and extension—these overall length measurements typically have a large error. With the idea of evaluating size variation more accurately, the 12^th^ body ring only was dissected from each specimen and measured for: (1) width (Fig. 2C) and (2) length (Fig. 2D) of the metazonite in dorsal view; and (3) the paranota lateral extension from a posterior view (Fig. 2E). Measurement of a single ring reduces the error, because a single diplosegmental ring is rigid and inflexible, and hypothetically linearly correlated to overall length. To control for a non-normal body size distribution, a natural logarithm to transform the raw measurements was used. Linear regressions were then used to evaluate potential correlations between the measurements and elevation.

*Cherokiageorgiana* exhibits a considerable diversity in coloration patterns throughout its geographical distribution. To evaluate this variation, pictures of the species taken from the specimens selected for the analysis and those observed on iNaturalist (available from https://www.inaturalist.org; accessed May, 2020) were included. Before including pictures from iNaturalist, identifications of the observations of *Cherokia* were confirmed by the authors (accessed on 18 May 2020). Afterwards the pictures were coded based on selection of one of three hue (red, orange, and yellow) and pattern groups (bimaculate, trimaculate, and striped), and scored. These pattern codes were then mapped onto the distribution of *Cherokia* to test if there is a correspondence with geographical areas and phylogenetic relationships.

## ﻿Results

### ﻿Selection of samples and Citizen science project

The citizen science campaign on social media received more than 100 responses from a Facebook and Twitter post. This resulted in 68 people who completed a Google form expressing their interest to participate in the project. Fifty people were then selected based on their location and proximity to areas previously not surveyed. Due to the limited number of kits available, sampling efforts were focused on the collection of millipedes in targeted localities in Georgia, Alabama and Tennessee. A total of 41 kits (Fig. 3) were shipped between the months of July and August of 2020 to participants who provided all the required information in the online form. From October 2020 to March 2021, a total of 23 live millipedes were received as a result of this project, and 13 of them were identified as *Cherokia* and included in the morphological and molecular analysis.

A total of 106 individuals from the genus *Cherokia* were included in the molecular phylogenetic analysis: 74 males, 31 females and one juvenile. Of these, 88% of the selected samples were previously deposited at the VTEC, and the remaining 12% corresponded to new samples obtained from the citizen science project.

### ﻿DNA extraction, phylogenetics analysis and species delimitation

The amplification and sequencing of DNA for the loci, COI, 12S, tRNA-Val and 16S, had a high rate of success, and only one specimen did not amplify (Suppl. material 1). For the locus fBox, the rate of success in amplification and sequencing was 96%, and for the loci EF1α and RNAPol2 that rate was considerably lower with 75% and 55% of the total sequences obtained. When amplifications and/or sequencing failed, amplifications were repeated up to three times using the same DNA extraction before discontinuing attempts.

**Figure 4.**
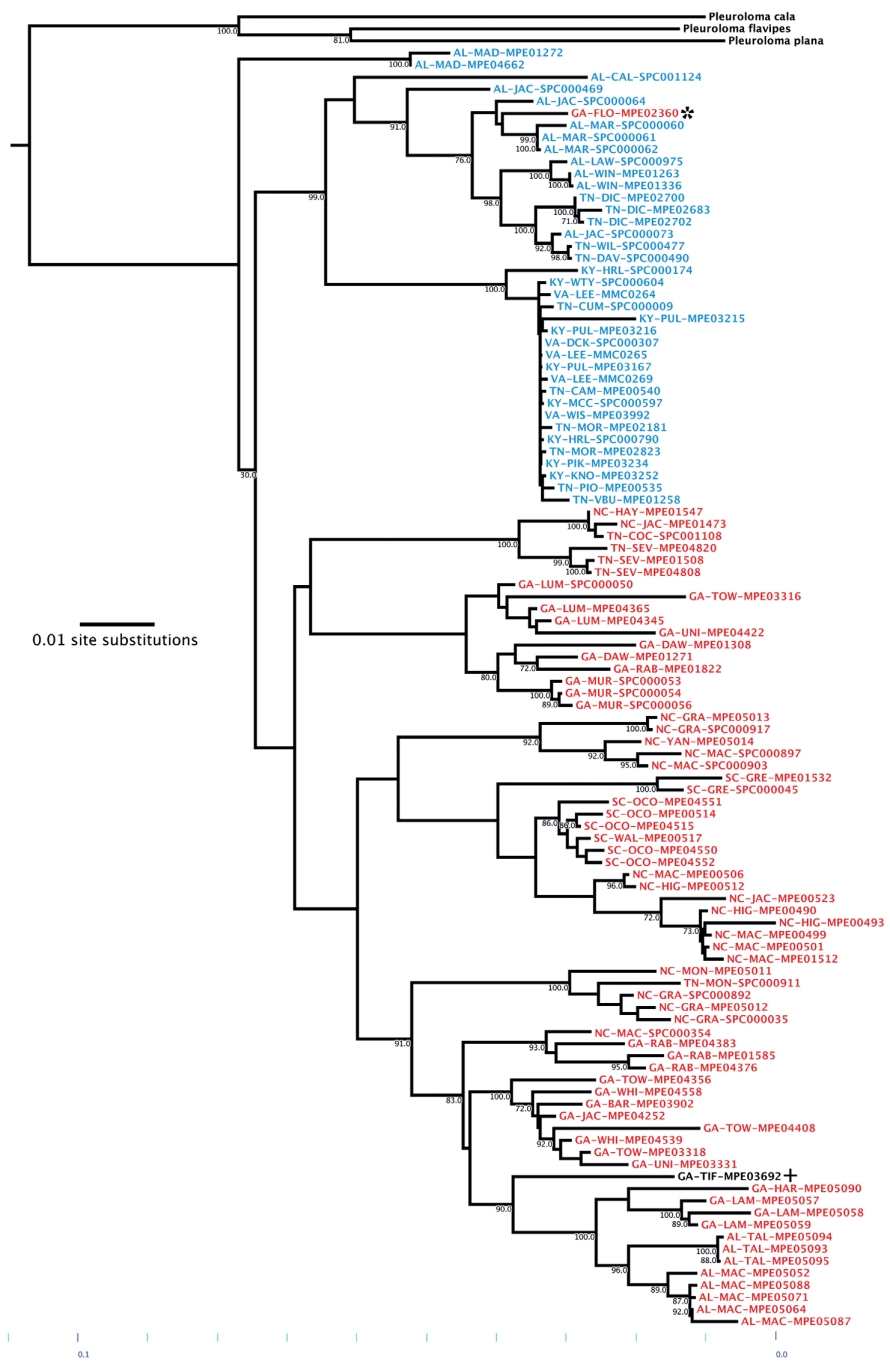
Phylogenetic reconstruction of the genus *Cherokia* Chamberlin, 1949. Terminals include the state, county and unique specimen code (i.e., AL-MAD-MPE01272). + Juvenile. * Outlier.

The multiple sequence alignment in MAFFT and inference of nucleotide evolution models in ModelFinder resulted in a 3865 bp concatenated matrix divided into six partitions and composed of 142 bp of 12S (TIM+F+G4 nucleotide evolution model), 82 bp of tRNA-Val (TIM+F+G4), 1081 bp of 16S (TIM+F+G4), 600 bp of COI (pos1 TN+I+G4, pos 2 TIM3+F+R2 and pos 3 TIM3+F+G4), 585 bp of EF1α (pos 1 & 2 TN+I+G4, pos 3 TIM3+F+R2 and intron GTR+F+I+G4), 978 bp of RNAPol2 (pos 1, 2, 3 & intron 1 TN+F+R2 and intron 2 TIM+F+G4), and 397 bp of fBox (pos 1 & 2 TN+I+G4 and pos 3 TIM3+F+R2). Of the 3865 nucleotide characters, 2726 corresponded to constant sites, 738 were parsimony-informative, and 401 were singleton sites. The average uncorrected pairwise distance for COI sequences between individuals from the same locality was 0.00470 (max. = 0.01644, min. = 0, σ = 0.005), and in total 0.07704 (max. = 0.12105, min. = 0, σ = 0.02742). The maximum uncorrected pairwise distance (COI) between *Cherokia* and *Pleuroloma* was 0.14740. The estimated phylogeny for *Cherokia* using the seven loci and the above-mentioned partitions and models is shown in Fig. 4.

**Figure 5.**
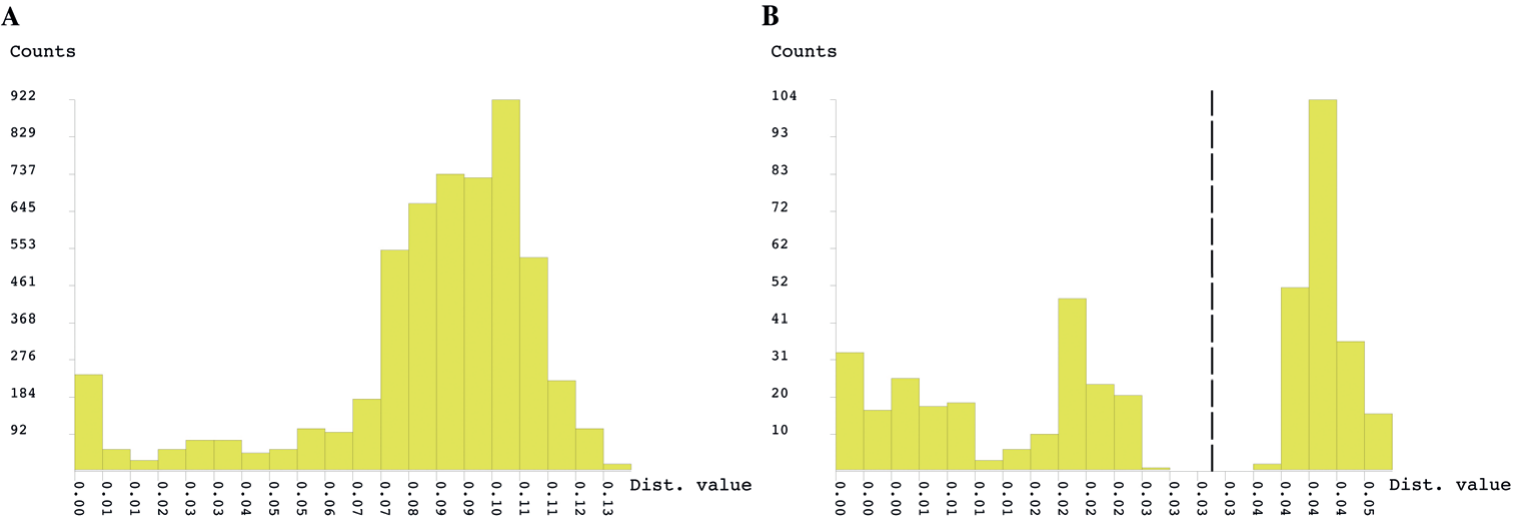
ABGD analysis results **A***Cherokia* Chamberlin, 1949 sequences, no barcode gap observed **B** simulated sequences, barcode gap marked by the dotted line.

The ABGD analysis included high-quality COI sequences for 105 specimens of *Cherokia* and excluded the sequences from the outgroup taxon *Pleuroloma*. The analysis was carried out on the ABGD web server using the Jukes-Cantor (JC69) substitution model and a relative gap width of 1.5X. The results of this analysis showed that a barcode gap does not exist in the COI sequences of *Cherokia* (Fig. 5A), and supports the model that all the individuals belong to the same group. Fig. 5B depicts what an expected histogram with a barcode gap present would look like; the dotted line marks the separation between the two groups and represents the likelihood of two species.

### ﻿Distribution mapping and morphological character analysis

**Figure 6.**
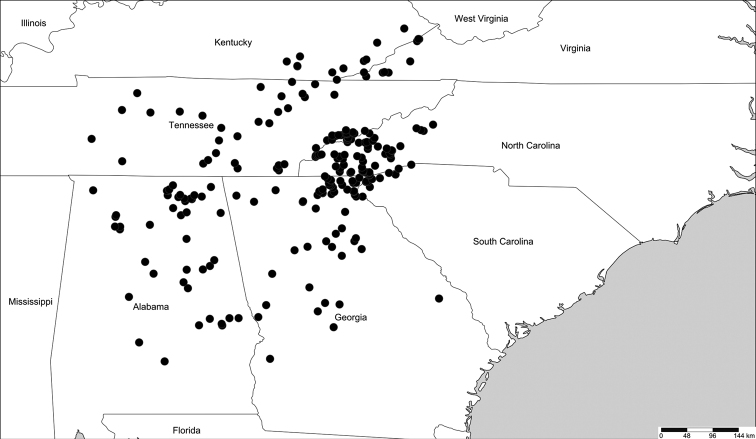
Geographical distribution of the genus *Cherokia* Chamberlin, 1949. Mapped using a set of 235 coordinates, from 848 individual records from Hoffman (1960), and natural history collections (VMNH, FSCA, VTEC).

A total of 201 reports were digitized and georeferenced from Hoffman (1960) (*N* = 103), the VMNH (*N* = 31) and FSCA (*N* = 67) natural history collections. Localities from the VTEC were obtained (already databased), thereby adding 222 *Cherokia* records to the database. The map for the geographical distribution of the genus *Cherokia* (Fig. 6) was constructed using 253 coordinates from localities representing 848 individuals. The geographical distribution includes seven states: Alabama, Georgia, Kentucky, North Carolina, South Carolina, Tennessee and Virginia. Ninety-six counties from throughout the aforementioned states have records of *Cherokia* individuals.

All of the adult individuals used for the phylogeny were included in the morphological analysis. The juvenile (Fig. 4; GA-TIF-MPE03692) was excluded due to lack of development in its morphological characters, which could have introduced unwanted outliers and substantial error in the data set. The measurement from the metazonal width had the greatest variation range (range = 6.0–9.3 mm, σ = 0.74, *N* = 105), followed by the paranotal extension (range = 1.25–2.17 mm, σ = 0.24, *N* = 105), and lastly by the metazonal length (range = 1.54–2.60 mm, σ = 0.20, *N* = 105).

**Figure 7.**
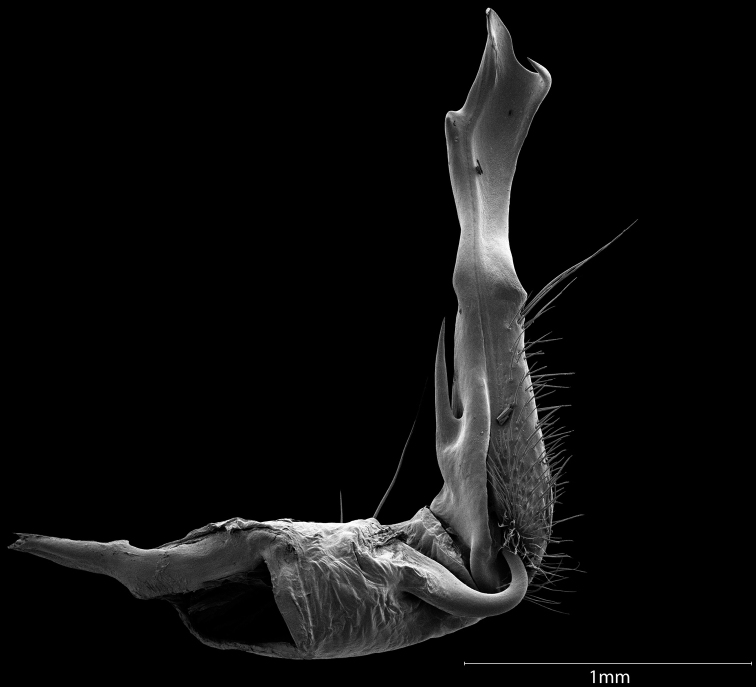
Scanning electron micrograph of a *Cherokiageorgiana* male gonopod. Medial view of specimen MPE04252 (VTEC).

**Figure 8.**
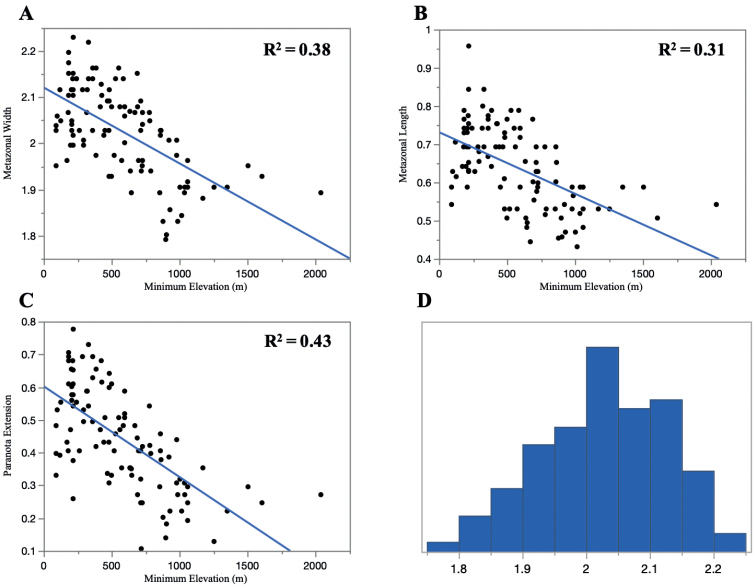
Linear regression of the elevation and body measurements **A** metazonal width **B** metazonal length **C** paranota extension and **D** Ln-transformed metazonal width distribution.

Once all the measurements were log-transformed, a linear regression analyzing the correlation between elevation and body dimensions were conducted for each of the respective measurements (Fig. 8). These analyses suggest that, in general, there is a negative correlation between the body measurements and the elevation; millipedes with smaller body sizes tended to be present in a higher elevation than those with a larger size.

**Figure 9.**
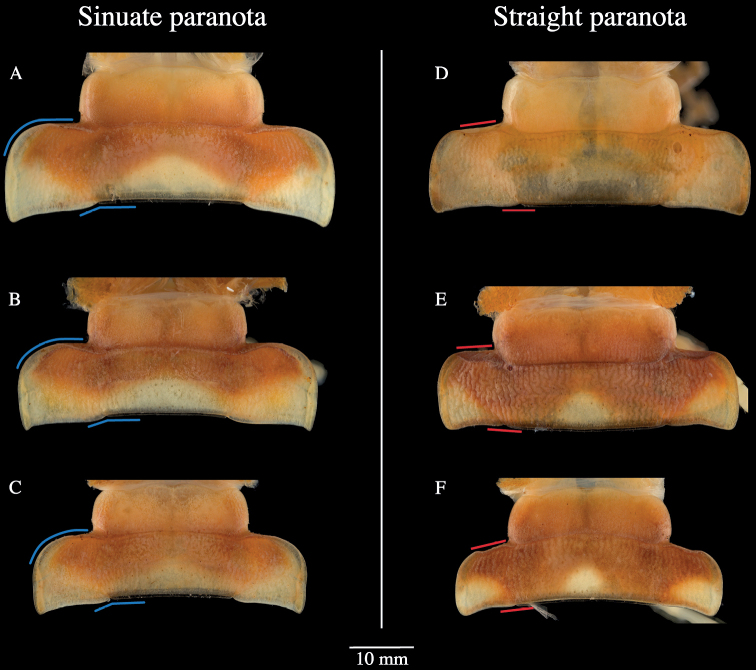
Variation in the paranota shape in *Cherokia* Chamberlin, 1949. Images of the 12th body ring of males (VTEC) showing sinuate paranota **A** SPC000060 (AL-MAR) **B** MPE01272 (AL-MAD) **C** MPE01336 (AL-WIN) or straight paranota **D** MPE02360 (GA-FLO) **E** MPE01308 (GA-DAW) **F** MPE01822 (GA-RAB). Blue and red lines denote the differences between the paranota shape.

The position of the scapulora as described in Hoffman (1960) (Fig. 2) could not be consistently discerned and objectively scored and was not included in this analysis. Nevertheless, a qualitative difference in the shape of the anterior border of the paranota was observed and generally showed two phenotypes for this character. The first phenotypic group includes a distinct sinuous curvature on the anterior border of the paranota, while the posterior paranotal corner protrudes backwards posteriorly beyond the margin of the posteromedial margin of the metazonite (Fig. 9A–C, blue lines). The second phenotypic group includes an almost straight anterior border, and the posterior corner is nearly aligned with the posteromedial margin of the metazonite (Fig. 9D, E, red lines).

The coloration analysis of *Cherokia* included a total of 124 images of individuals identified as *Cherokia* on iNaturalist. The identifications of *Cherokia* observations on iNaturalist were confirmed by the authors based on the diagnosis below. The pictures were coded using the three colors (red, orange and yellow), and three patterns (bimaculate, trimaculate, and striped). Most of the individuals exhibited only one of the colors, and a smaller proportion of them exhibited two. The color white was only observed present while in combination with another color (i.e., white and orange), while the other colors were present by themselves or with another.

**Figure 10.**
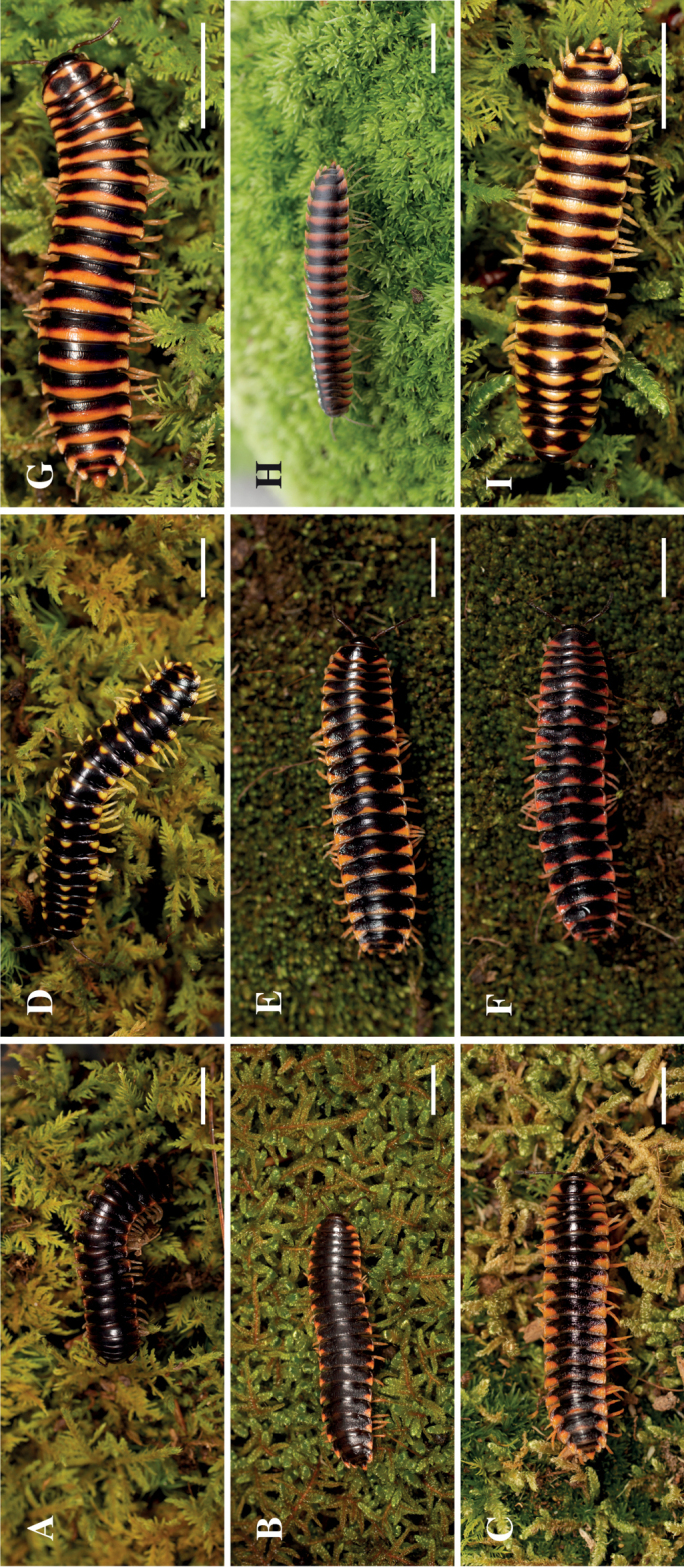
Coloration patterns observed in *Cherokia* Chamberlin, 1949. Bimaculate **A** MPE01512 (NC-MAC) **B** MPE04252 (GA-JAC) **C** MPE02181 (TN-MOR), trimaculate **D** MPE01508 (TN-SEV) **E** MPE01225 (TN-VBU) **F** MPE01227 (TN-VBU), and striped **G** MPE00505 (NC-HIG) **H** MPE04515 (SC-OCO) **I** MPE00501 (NC-HIG). All specimens are from the VTEC. Scale bars 10 mm.

In the bimaculate pattern, a spot of color was present laterally on each paranota (there are two paranota per ring) with the center lacking pigmentation (Fig. 10A–C). The trimaculate pattern, is characterized by a coloration spot on each paranota in addition to a middorsal spot on the ring. The middorsal or paranotal spots had different sizes and could be one of three shapes: a circle, oval, or a triangle (Fig. 10D–F). The striped pattern is where a color band is on the posterior margin of the body ring that runs from one paranota to the other. The band could have various thicknesses, and in some cases an apparent superposition of the trimaculate pattern was evident atop the banded pattern (Fig. 10G–I). There was no clear relationship between geographical distribution and the color or patterns; in some cases, syntopic individuals of *Cherokia* from the same locality exhibited different coloration patterns.

### ﻿Taxonomy

**Family Xystodesmidae Cook, 1895**

**Subfamily Rhysodesminae Brolemann, 1916**

#### Tribe Rhysodesmini Brolemann, 1916

##### ﻿Genus Cherokia

Taxon classificationAnimaliaPolydesmidaXystodesmidae

Chamberlin, 1949

9CCD7B56-824C-50B2-B2B9-9056BBD5B2B2

###### Type species.

*Cherokiageorgiana* (Bollman, 1889)

##### ﻿ Cherokia georgiana

Taxon classificationAnimaliaPolydesmidaXystodesmidae

(Bollman, 1889)

B414B56C-6304-5C84-AA73-A586584CD760

Fontaria georgiana Bollman, 1889a: 344. MALE HT (United States National Museum, USNM). United States: Georgia, Bibb County.
Fontaria tallulah Bollman, 1889a: 344. FEMALE HT (USNM). United States: Georgia, Habersham County. Synonymized by Hoffman, 1950b: 23.
Mimuloria furcifer Chamberlin, 1940a: 282, fig. 1. MALE HT (USNM). United States: North Carolina, Buncombe County. Synonymized by Hoffman, 1950b: 23.
Mimuloria georgiana – Loomis 1943: 402.
Dynoria parvior Chamberlin, 1947: 10, fig. 4. MALE HT (USNM). United States: Georgia, Union County. Synonymized by Hoffman, 1950b: 23.
Cherokia georgiana – Chamberlin 1949a: 3.
Cherokia georgiana georgiana Hoffman 1960: 240, figs 3d, 4e, 5a, 6, 7. syn. nov.
Mimuloria ducilla Chamberlin, 1939: 7, fig. 12. MALE HT (USNM). United States: North Carolina, Jackson County.
Mimuloria georgiana (nec Bollman, 1889) – sensu Loomis, 1943: 402.
Cherokia georgiana ducilla Hoffman 1960: 255, figs 3b-e, 4f, 5b, 6, 7. syn. nov.
Cherokia georgiana latassa Hoffman, 1960: 257, figs 3a, c, 4a–e, 5c, d, 7. MALE HT (USNM). United States: Tennessee, Warren County. syn. nov.

###### Note.

For a complete taxonomic listing, see Means et al. (2021b), Suppl. material 1.

###### Diagnosis.

Adults in the genus *Cherokia* are distinct from other rhysodesmine genera based on the following combination of characters: **Body rings**: dorsal surface of the metazonites with a noticeably wrinkly texture. Paranota horizontal and wide, with little downwards curvature, making the body appear flatter than other rhysodesmines. **Gonopods**: Telopodite sublinear in shape (Fig. 7), not distinctly curved or twisted as in the Apheloriini. Telopodite with a cingulum. Acropodite with its apex appearing flat and truncated. Telopodite with a long acicular prefemoral process; not a stout, curved prefemoral process nor wholly lacking as in the Apheloriini. **Cyphopods**: receptacle absent. **Coloration**: yellow to red hues in bimaculate, trimaculate and striped patterns (Fig. 10). Yellow trimaculate is the most frequent color morph (Fig. 1).

## ﻿Discussion

The previously reported geographical range of *Cherokia* sensu Hoffman (1960), included six states, 43 counties and 93 localities. Here we report the presence of *Cherokia* in a seventh state (Virginia) and 53 new counties, for a total of 160 new localities where specimens of the genus have been collected. In prior systematic analyses of the millipede family Xystodesmidae, *Cherokia* was represented by three individuals, sequenced for six genes (Means et al. 2021b). Here, to address species boundaries in greater detail, we increased this number to 106 individuals sequenced for seven genes, for a total of 450 sequences and 3865 base pairs of DNA. These sequences were used to infer a phylogeny (Fig. 4). Of the seven loci amplified and sequenced for the phylogenetic reconstruction of the genus *Cherokia*, RNAPol2 was less successful than others in terms of amplification (presence of bands on electrophoretic gels) and sequencing (low quality reads: phred scores > 20). The presence of stop codons in RNAPol2 sequences, despite viewing in six alternative reading frames, is unexpected and may indicate that it is a recent pseudogene. However, the relatively lower success in sequencing of this locus does not appear to affect the general topology of the phylogeny.

Based on the molecular phylogeny, *Cherokia* is a monophyletic taxon (Fig. 4) sister to *Pleuroloma*. There is a clade formed by two individuals from the same locality (Monte Sano State Park, Madison Co., Alabama) that is sister to the remaining ones. Three statistically well-supported clades are present and subtended by long branches; however, the other individuals in the phylogeny are paraphyletic with respect to these clades and are not reciprocally monophyletic with them. In general, individuals from the same locality or nearby localities grouped together. Individuals from Kentucky and Virginia occur together with some individuals from Tennessee in a clade with very short branches. This block of individuals corresponds with the northeastern limit of the geographical range of the genus, and to the Cumberland Mountain Thrust Block region, a mountainous and complex region lying between the dissected Appalachian Plateau to the west and the Valley and Ridges to the east. This region also houses a clade of millipedes in the genus *Brachoria* Chamberlin, 1939 with similarly very shallow genetic divergences as *Cherokia* (Marek 2010). These shallow branches in *Cherokia*, as in *Brachoria* (Marek 2010), may represent relatively more recent and/or rapid diversification in the area, and may be due to shared mechanisms of regional diversity, or be associated with mimicry evolution in the area. *Cherokia* is a known participant of Müllerian mimicry in the region (Marek and Bond 2009; Marek 2010).

The morphological characters evaluated by Hoffman (1960) were reexamined with new measurements and compared to geographical variables (i.e., elevation) and the phylogeny. The measurements taken from the 12^th^ body ring and its inverse linear correlation with elevation showed that, in general, individuals of *Cherokia* with smaller body size and shorter paranota tend to be present at higher elevations than those with a larger size and longer paranota (Fig. 8). While the new measurements showed the same distribution as Hoffman (1960), the variation appears to be clinal, and not discordant variation with abrupt changes that would be expected to correspond to species boundaries. Many terrestrial invertebrate taxa show smaller body sizes at higher elevations, but the converse has also been observed (Hodkinson 2005). Smaller *Cherokia* at higher elevations may be associated with resource limitation as has been implicated in other terrestrial invertebrate groups (Hodkinson 2005). Alternatively, the smaller body sizes may be associated with body shape differences linked to burrowing efficiency in different leaf-litter substrates at higher elevations (e.g., there is a greater diversity and abundance of evergreen trees at higher elevations).

The results of the ABGD analysis showed a congruent pattern where genetic distances are continuously distributed and no barcode gap exists (Fig. 5A). This shows that there are no clear genetic clusters indicative of a barcode gap for distinct species or subspecies (Fig. 5B). While sampling effort may affect ABGD analyses, our dataset of 106 specimens uniformly sampled from across the distribution of the genus supports the hypothesis of a single, widespread species.

**Figure 11.**
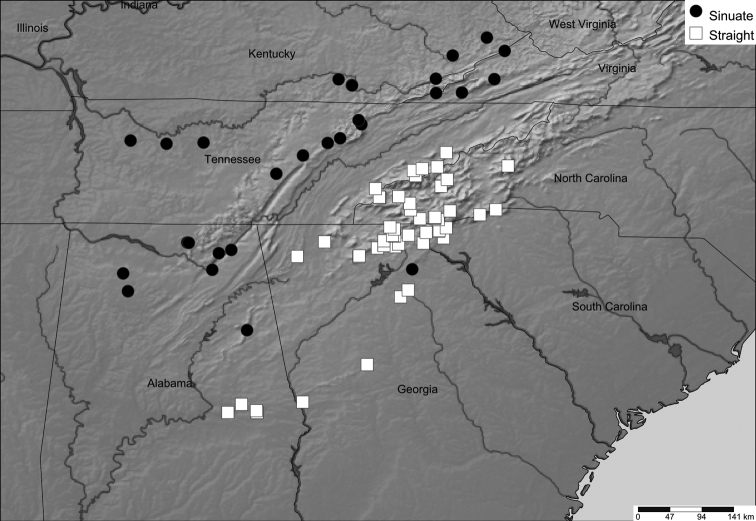
Geographical distribution of *Cherokia* Chamberlin, 1949, showing the two types of paranota shape. Map includes specimens used for the morphological analysis and deposited at VTEC (*N* = 105).

The position of the scapulora (sensu Hoffman 1960) was not a useful character, due to the difficulty of distinguishing its two states from each other (marginal and submarginal); perhaps this is due to its continuous nature, as is the case with the body size characters above (Fig. 8). During the examination of this character, we observed that the two states (marginal and submarginal) were not phylogenetically or geographically concordant. As described above (Fig. 9), the anterior margin of the paranota roughly grouped into two distinguishable shapes: sinuate or straight. To evaluate the relevance of this newly discovered character, its geographical distribution was mapped (Fig. 11).

The geographical distribution shows that the individuals with sinuate paranota generally tend to be located in the western part of the Appalachian region, while the individuals with a straight paranota are located in the eastern part. This separation appears to correspond to the Tennessee River Valley and the geological barrier that it represents for the genus, and other co-distributed taxa (e.g. *Nannariawilsoni* species group; Hennen et al. 2022). However, in the southern part of the geographical distribution of *Cherokia*, especially in the state of Alabama, both shapes of the paranota overlap and no clear geographical separation was observed (Fig. 11). When this character was traced on the phylogeny of the genus, most individuals in one clade exhibited straight paranota (Fig. 4, blue), while the other clade (and two individuals from Monte Sano State Park, Alabama) possessed sinuate paranota (Fig. 4, red). One individual in the phylogeny and geographical distribution appears as an outlier for the general trend of this character (Fig. 4, GA-FLO-MPE03260*). Although a qualitative character and correlated with metazonite width (p = 0.0001), in some cases it is difficult to distinguish straight versus sinuate, and the variation appears to be clinal. In contrast with the scapulora and color characteristics, this character is largely concordant with the phylogeny, but in itself as a single character, insufficient for species or subspecies delimitation.

**Figure 12.**
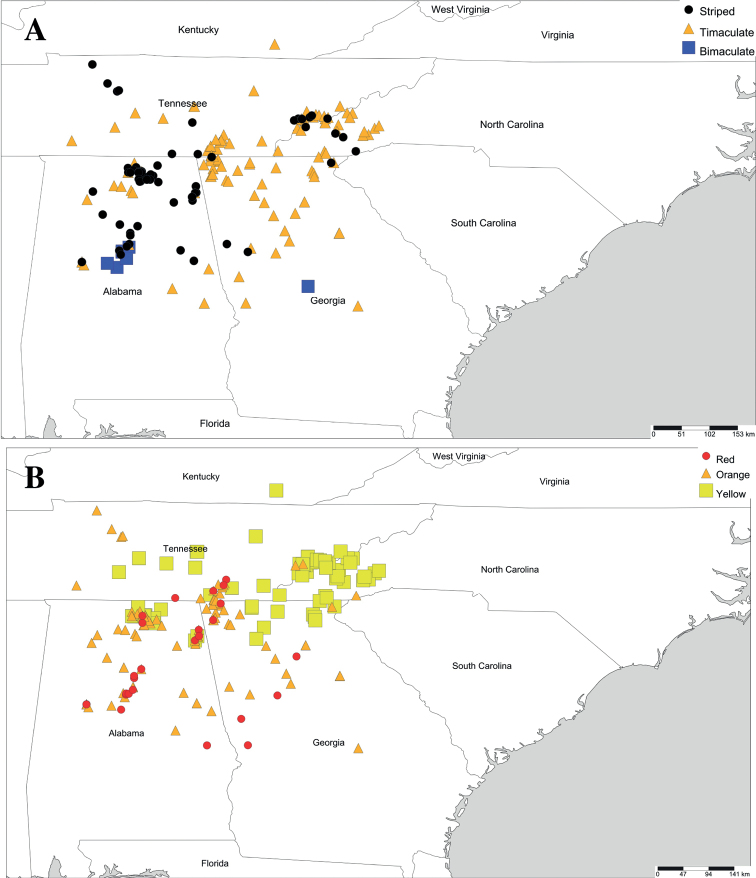
Geographical distribution of *Cherokia* Chamberlin, 1949 vs. coloration patterns **A** patterns **B** colors. Mapped using iNaturalist pictures reported for *Cherokia* (*N* = 124).

The coloration patterns were plotted on a map to assess concordance with the geographical distribution. Fig. 12 shows the distribution of the patterns (bimaculate, trimaculate, or striped), and the colors (red, orange or yellow). Some localities have all three types of patterns and/or colors—in contrast with Hoffman’s (1960) supposition that each coloration is geographically isolated. Nearly all possible combinations of colors and patterns were observed, but the trimaculate yellow color morph was the most common (both in frequency of individuals and geographical area). The bimaculate pattern was only observed with an orange hue (the bimaculate orange color morph, Fig. 10A–C). Fig. 12 shows that neither the pattern (bimaculate, trimaculate, striped) nor the colors (red, orange, yellow), have any clear geographical association. [Note that the number of geographical data points that were used for these maps (Fig. 11) were greater (*N* = 124) than the one used for the phylogenetic analysis (*N* = 106). Because the number of images available for the specimens actually used in the phylogeny was relatively small (*N* = 26) and limited the scope of inference, iNaturalist reports for *Cherokia* were also included in this section.] Perception of color can be affected by the observer, lighting conditions, veiling conditions, and distance, thereby adding error to the evaluation of this character (Endler 1990). In the future, a less error-prone and less human-centric technique should be implemented to obtain more accurate coloration data such as using a spectrometer and incorporating the visual systems of the predators of *Cherokia* (likely avian) to evaluate the coloration according to the perceivers’ eyes.

The use of citizen science as a tool for obtaining and analyzing data has been successfully demonstrated by various research groups. The Cornell Lab of Ornithology, for example, has developed multiple projects involving amateur ornithologists and the general public for around two decades. Data obtained from those initiatives have been published in several peer-reviewed research papers in various journals (Bonney et al. 2009). The small-scale citizen science project that was made as part of this research demonstrated that it is an effective method to obtain samples from remote and inaccessible localities, or in special situations such as the SARS-CoV-2 pandemic. Although the first response to the initiative was highly positive, follow-up contact with the interested participants was more difficult and less successful. The number of people who shipped samples back to us (*N* = 12) corresponds to around the 30% of the kits shipped to selected participants (*N* = 41). Improved communication with the participants, and a more structured timeline will be needed to increase the overall success of this initiative in future projects. Nonetheless the citizen science project offered an impactful opportunity to share the research with a broader community.

## ﻿Conclusions

Morphological characters showed clinal variation and a direct relationship with geographical distribution and elevation, but not with the phylogeny. Coloration was highly variable and did not accord with neither geography nor phylogeny. The phylogeny recovered *Cherokia* as a monophyletic taxon, and the ABGD species delimitation test showed no barcode gap supporting the existence of multiple species. The molecular and morphological evidence showed that *Cherokia* is a monospecific genus with the sole species *Cherokiageorgiana* being geographically widespread and highly variable in its morphology.

## Supplementary Material

XML Treatment for Cherokia

XML Treatment for Cherokia georgiana

## Supplementary materials

Supplementary material 1 Specimens used in the phylogenetic analysis Data type List of taxa and NCBI accession numbers Explanation note List of specimens used in the phylogenetic analysis, with their localities and NCBI accession numbers. https://binary.pensoft.net/file/703824This dataset is made available under the Open Database License (http://opendatacommons.org/licenses/odbl/1.0/). The Open Database License (ODbL) is a license agreement intended to allow users to freely share, modify, and use this Dataset while maintaining this same freedom for others, provided that the original source and author(s) are credited.Luisa Fernanda Vasquez-Valverde, Paul E. Marek

Supplementary material 2 *Cherokiageorgiana* specimens examined Data type List of taxa Explanation note List of *Cherokiageorgiana* specimens examined from literature and natural collections. https://binary.pensoft.net/file/703825This dataset is made available under the Open Database License (http://opendatacommons.org/licenses/odbl/1.0/). The Open Database License (ODbL) is a license agreement intended to allow users to freely share, modify, and use this Dataset while maintaining this same freedom for others, provided that the original source and author(s) are credited.Luisa Fernanda Vasquez-Valverde, Paul E. Marek

Supplementary material 3 Individual gen trees Data type Phylogenetic Explanation note Individual gene phylogenies of *Cherokiageorgiana*. https://binary.pensoft.net/file/703826This dataset is made available under the Open Database License (http://opendatacommons.org/licenses/odbl/1.0/). The Open Database License (ODbL) is a license agreement intended to allow users to freely share, modify, and use this Dataset while maintaining this same freedom for others, provided that the original source and author(s) are credited.Luisa Fernanda Vasquez-Valverde, Paul E. Marek
